# Infant and early childhood mental health Endorsement: Participant reports and perceptions

**DOI:** 10.1002/imhj.70056

**Published:** 2025-10-29

**Authors:** Angela Tomlin, Ashley N. McCormick, Ann Marie Martin, Anicia Battles, Lauren Moberg, Julia Gutierrez‐Albrecht, Nilou Pariborzi, Ashley McGinn, Sarah Brown, Krista Longtin, Diana Morelen

**Affiliations:** ^1^ Indiana University School of Medicine Indianapolis Indiana USA; ^2^ Alliance for the Advancement of Infant Mental Health Southgate Michigan USA; ^3^ Minnesota Association for Children's Mental Health Saint Paul Minnesota USA; ^4^ Sanilac Great Start Collaborative Sandusky Michigan USA; ^5^ INfancy Onward Indianapolis Indiana USA; ^6^ East Tennessee State University Johnson City Tennessee USA

**Keywords:** infant/early childhood mental health, reflective supervision and consultation, workforce development

## Abstract

There is agreement across infant and early childhood fields that infant and young children's development depends on quality caregiving, which in turn requires a competent and well‐supported workforce. This paper includes results of 3 qualitative questions from an international survey (U.S. and Australia, *n* = 911) of holders of an Infant or Early Childhood Mental Health Endorsement credential that documents their knowledge and skills. The U.S.‐based research team used a qualitative content analysis approach to code the responses. Benefits reported included increased knowledge of infant and early childhood mental health, recognition of expertise, and the opportunity to participate in a professional network. Participants also identified barriers to earning Endorsement, including time and money. Themes were analyzed with post hoc Chi Squares by participant demographic characteristics. Asian participants were more likely to report that Endorsement provided personal credibility and recognition and brought validity to the field. American Indian participants were more likely to indicate that Endorsement validates the professional field. Participants from more established associations were more likely to report concerns about the costs of Endorsement. Results are discussed in the context of ongoing system changes that can increase diversity in leadership and the overall infant and early childhood workforce.

There is agreement across infant and early childhood fields that infant and young children's development depends on quality caregiving, which in turn requires a competent and well‐supported workforce (Kwon et al., 2021; Morelen et al., 2020; Pavkov & Wells, 2023). Initiatives to identify competencies to build and support specific sectors of the workforce have been developed for fields including early education (*ZERO TO THREE*, 2018 *)*, early care (American Federation of State, County and Municipal Employees, et al., Unifying Framework, 2020; LeMoine et al., 2023), and home visiting (HARC, n.d.; Walsh et al., 2020). Although the workforce serving infants and young children and families is diverse and serves families in a range of different ways, it has been noted that similarities underlie both the work and the challenges inherent in the work (Carolan & Fishel, 2023). As a result, there are also some initiatives intended to support a broader range of providers. Some are state‐specific, such as the Illinois Competency‐Based Assessment System (Dietz and Ernst, 2023), and others are offered nationally, including ZERO TO THREE's P‐5 Competencies (Dean et al., 2019; Zero to Three, 2018). The credential developed by the Michigan Association for Infant Mental Health and now administered by the Alliance for the Advancement of Infant Mental Health (Alliance), the *Endorsement for Culturally Sensitive, Relationship‐focused Practice Promoting Infant Mental Health* (Endorsement) may be considered the most widely disseminated framework for developing the infant and early childhood workforce, evidenced by its use in 36 states and 2 countries, crosswalks to major evidence‐based programs serving infants and young children and families such as Healthy Families, Child Parent Psychotherapy, and Circle of Security and appearance as a required element in some federal grant proposals (Morelen et al., 2020; Shea et al., 2022; Weatherston et al., 2009).

Launched in 2002, with revisions in 2017, 2021, 2022, 2023, and 2024, the Endorsement system includes a set of competencies (*Competency Guidelines for Endorsement for Culturally Sensitive, Relationship‐Focused Practice*) and several types of Endorsement pathways, which are professional credentials that can be earned by a wide range of providers who serve children from birth to 6 years and their families (Funk et al., 2017). Participation in Endorsement is designed to demonstrate the individual's commitment to professional development and denotes expertise in infant and/or early childhood mental health within one's scope of professional practice. Earning the Endorsement credential requires demonstration of knowledge, training, work experience, and ethical values through preparation of an application, with some Endorsement pathways also requiring participation in reflective supervision or consultation (RSC; i.e., a form of professional development that is relationship based and attends to the professional's emotional response to the work) and passing a written examination (Meuwissen et al., 2024; Weatherston et al., 2009).

**Key Findings**

- Participants in an infant and early childhood mental health credential reported benefits including increased personal recognition, opportunities for networking with others, and validity for the field/workforce.
- Barriers to participation include time to complete the application, costs to participate, and ability to access reflective supervision/consultation.
- Participant self‐reported race and work experience and the longevity of the association that they belonged to were related to reported experiences with the process.

**Relevance for Infant and Early Childhood Mental Health**

High‐quality care and services are essential to infant young children's development and wellness; therefore, a well‐trained and supported workforce is a priority. This study examines an effort to ensure a competent infant and early childhood mental health workforce through a professional credential. The results help us understand participants’ experiences taking part in the system and provide suggestions for improving the system to be more equitable.

Over time and increasingly with intentionality, the infant and early childhood workforce has grown and become more diverse (Dealy et al., 2023). Despite diversity spanning cultural, linguistic, racial, and ethnic differences within many sectors, leadership diversity has lagged in infant and early care fields, leading to discussions about equity in practices and systems, including licensing and credentialing processes such as the Endorsement process. Examples of responses to date include removing the requirement for submitting official transcripts, translating Endorsement materials to Spanish, providing accommodations to test takers whose first language is other than English, removing the degree requirement for some applicants, and developing a new category to allow a larger range of people to qualify to provide RSC within the system. Despite this work, challenges remain regarding what would make this and other credentialing processes more accessible, equitable, meaningful, and effective from a workforce development perspective.

Credentialing systems increasingly impact infant and early childhood fields in ways that may include influencing training and curriculum at many levels, shaping personal and agency budgets, and, at times, serving a gatekeeping function to those seeking to join the workforce. Given that Endorsement is the most widely disseminated credential in the IECMH fields, work is needed to understand its strengths and limitations, including those that may contribute to inequities. Previous efforts to evaluate Endorsement have yielded some data on benefits and barriers. McCormick & Eidson (2020) reviewed evidence for the benefits of Endorsement, including a survey of nearly 1300 infant family professionals from 14 states (Krysik et al., 2019). In this study, close to 90% of participants rated Endorsement as highly beneficial or beneficial in their ability to understand infant mental health, promote infant mental health, and promote social‐emotional development in babies and toddlers. Similarly, 115 participants involved in the Endorsement experience in Virginia reported benefits such as the opportunity for professional development and connections to other professionals (Dye et al., 2023). Time and costs associated with accessing RSC were cited as barriers to Endorsement. A recent evaluation assessed the experiences of front‐line professionals and leaders (directors and supervisors) endorsed as Infant Family Associates, primarily representing early care and education (AIR, 2023). Broadly, the results indicated a positive view of receiving training leading to Endorsement. Participants strongly agreed that being endorsed will help in their career (82%); help in their work with families (64%); and increase their likelihood of staying in the field (62%).

A recent in‐depth survey regarding experiences and perceptions of participation in the Endorsement system and process was conducted by a team of interdisciplinary researchers and IECMH system leaders. The survey is one part of a broader effort to improve the Endorsement process with attention to the needs of the increasingly diverse workforce. This survey included the broadest participation of endorsed professionals studied to date, increasing the potential generalizability of the results compared to previous work. All US state infant mental health associations that were actively offering Endorsement at the time, as well as Western Australia, were asked to invite all endorsed professionals in their state/region to participate in the survey. Participants represented multiple sectors of the infant and early childhood workforce and holders of all 8 pathways of Endorsement (e.g., Infant and Early Childhood Family Associate, Infant and Early Childhood Family Specialist, Infant and Early Childhood Mental Health Specialist, and Infant and Early Childhood Mentor in Clinical, Research, and Policy). The present study will describe the results of participant responses to 3 open‐ended questions about their experiences with earning Endorsement.

## 1 METHODS

### 1.1 Participants

Infant mental health associations in the US (33 states) and Western Australia (1) were asked to invite endorsed professionals to participate in an electronic survey. Of 1800 providers currently holding Endorsement worldwide at the time of the survey, 911 responded. Participants identified as predominantly female (97%) and “White or Caucasian” (75%). Most had advanced degrees (72% with master's or above), and the average work experience in the field was 11.5 years. Employment sector experience was broad (e.g., community mental health 19%, home visiting 20%, early care/education/Head Start 40%, administration/policy 21%). See Table 1 for participant demographics.

**TABLE 1:** Participant demographics.

	*N* = 911	Q1 *n* = 494	Q2 *n* = 428	Q3 *n* = 340
Gender, *n* (% Female)	828 (91)	476 (96)	415 (97)	332 (98)
Race and Ethnicity, *n* (%)				
American Indian/Native American	8 (1)	6 (1)	4 (< 1)	2 (< 1)
Asian	22 (2)	11 (2)	14 (4)	12 (4)
Black	76 (9)	39 (8)	36 (9)	22 (7)
Latine (any race)	98 (12)	60 (12)	43 (10)	43 (13)
Pacific Islander/Native Hawaiian	5 (1)	2 (< 1)	2 (< 1)	1 (< 1)
White	636 (75)	371 (75)	320 (76)	259 (76)
Years of Experience, *n* (%)				
1–3	36 (4)	19 (4)	14 (3)	12 (4)
3–10	230 (27)	139 (28)	101 (24)	66 (19)
10–20	304 (36)	173 (35)	159 (37)	135 (40)
20+	280 (33)	161 (33)	152 (36)	127 (37)
Education, *n* (%)				
High School/GED	3 (< 1)	1 (< 1)	2 (< 1)	1 (< 1)
Some college/associate's degree	61 (7)	34 (7)	23 (5)	22 (7)
Bachelor's degree	172 (20)	103 (21)	73 (17)	62 (18)
Master's degree	527 (62)	316 (64)	278 (65)	224 (66)
Doctoral degree	86 (10)	39 (8)	51 (12)	31 (9)
Alliance establishment				
Newer (less than 10 years)	446 (53)	258 (52)	211 (49)	168 (50)
Established (more than 10 years)	401 (47)	234 (48)	216 (51)	171 (50)
Endorsement category				
Infant/early childhood family associate	218 (24)	132 (27)	83 (19)	85 (25)
Infant/early childhood family specialist	253 (28)	148 (30)	132 (31)	103 (30)
Infant/early childhood mental health specialist	245 (27)	137 (28)	123 (29)	95 (28)
Infant/early childhood mental health mentor	154 (17)	89 (18)	95 (22)	63 (19)
Alliance region				
International	9 (1)	7 (1)	8 (2)	2 (< 1)
Northeast	126 (14)	72 (15)	64 (15)	52 (15)
Midwest	236 (26)	135 (27)	126 (29)	84 (25)
South	259 (28)	143 (29)	119 (28)	93 (27)
West	217 (24)	135 (27)	110 (26)	108 (32)
None reported	64 (7)	2 (< 1)	1 (< 1)	1 (< 1)

### 1.2 Measures

The *Perceptions of Endorsement Survey* was created for this study by the participants of an Alliance Task Force examining the Endorsement process. The task force was composed of Alliance leaders and team members representing community voice and researchers across multiple states and agencies/institutions. The survey's purpose was to learn more about endorsed participants’ experiences with the Endorsement process to identify what is working well and to identify potential areas for improvement. This project is part of ongoing efforts to expand and diversify the IECMH workforce, including identifying and removing barriers to participation in Endorsement.

The complete survey included 3 main sections: participant demographics, a set of structured questions with Likert scale response options, and open‐ended items. Participant demographic information gathered included identified gender, race, ethnicity, languages spoken, educational attainment, work experience, work sector, communities served, Endorsement status, and specific Association membership. Participation in Endorsement by states and other entities has accrued gradually between 2002 and the present; at the time of the data collection, Endorsement was 20 years old. Therefore, we were interested in variation in the length of Association participation in Endorsement as a factor in participant experience. We dichotomized the Associations into two groups with more or fewer than 10 years' longevity, as this represented the mid‐point of possible years' participation. This division also resulted in 2 fairly even groups. The structured questions explored participants’ views of the impact of Endorsement on their work, including knowledge, skill, and professional opportunities, descriptions of their experiences accessing RSC, employer support for the process, and potential barriers. The survey also included open‐ended questions (1) How has earning Endorsement affected your work? (2) What would you tell others about why they should earn Endorsement? and (3) What else would you like us to know? The present study will examine the results of these 3 open‐ended questions.

### 1.3 Data analysis

A mixed methods approach was utilized for the present study. Qualitative data domains were identified using an inductive multiple‐round qualitative content analysis approach (Vaismoradi et al., 2013). Authors who participated in the coding process all held Endorsement, worked in the field of IECMH in some capacity, were members of an infant mental health association, and participated in an Alliance research affinity group on Endorsement. The majority of these authors did not have experience participating in research. Therefore, the coding process reported below was proposed by one member of the coding group in consultation with another author. The proposed coding process was presented to the other coding authors for discussion prior to adoption. The group met throughout the coding and review process to discuss progress and ensure consistency.

Authors worked in pairs to identify themes, assign responses to themes, and select representative responses for the study's three open‐ended questions. Each author independently reviewed the responses to identify themes. Pairs of authors came to a consensus on one set of themes via discussion. After the themes were identified, each author independently determined if a response should be coded into each theme. Each response was kept intact so that it could be categorized into one or more themes. After each author coded all responses, pairs reviewed and discussed the responses. The group determined that pairs would review items until they reached at least 80% consensus within a pair of raters (O'Connor and Joffe, 2020). During the consensus process, authors recognized there were limited disagreements. After discussion with the full group, it was determined that once an 80% agreement level was met, pairs could adopt one author's responses for the remaining items, yielding 100% agreement. All 3 groups used this option. During this process, some themes were further combined by agreement of the two raters.

In order to explore potential differences in how respondents answered the open‐ended questions, a series of chi‐squares was conducted between selected participant demographic characteristics (race and ethnicity, years of experience, education, sector affiliation, Endorsement category, and length of association establishment) and identified themes. Effect sizes are reported using phi or Cramer's V. In line with recommendations by Perneger (1998) and Rothman (2014), we did not use Bonferroni's adjustment for multiple tests. Throughout, a *p*‐value of 0.05 was set as the a priori threshold for statistical significance.

## 2 FINDINGS

### 2.1 Qualitative findings

There were three open‐ended questions with a total of 911 respondents. Data analyses revealed five major themes for the question, “what would you tell others about why they should earn Endorsement,” four major themes for the question, “how has Endorsement influenced your work,” and six major themes for the question, “what else would you like us to know.” The next section reports the results of themes identified in each section from most to least cited. Please refer to Table 2 for a summary of themes and sample quotes.

**TABLE 2:** Themes and sample quotes (*N* = 911).

Question	*N* (%)	Theme	Quote
How has Endorsement influenced your work?	494 (54)		
	178 (36)	Learning, growth, and improved knowledge	*Stumbling across the field of IMH, led me to endorsement and it's impossible to ever go back to the lens I had previously. It has opened up to new and expansive ways of thinking, seeing and understanding families that has been a game changer. My relationships with families has forever been altered, and they in turn have different relationships with their children*. *It has helped me have a deeper understanding of how infants and toddlers are affected by mental health. It has made a considerable impact on how I provide care to those in my classroom*. *It has directly impacted my ability to support professionals in increasing their ability to support early mental health in infants/young children/families they serve*. *While I had a MSEd degree in child development/early childhood, the full experience of the Endorsement process did change the way I view the world—quite profoundly in concert with my RS experience*. *Endorsement has influenced my work in a significant way. It gave me a language and deeper understanding of relationship based work*. *Becoming endorsed put me in a network of professionals who I have learned a great deal from*.
	157 (32)	Increased credibility and recognition as an expert	*Endorsement reflects the level of commitment I have taken to be trained and skilled in providing the most appropriate services to infants, young children, and their families*. *It has allowed me to showcase my expertise in IECMH which has led to additional leadership roles, being sought out as a trainer and TA provider for other colleagues or communities*. *Helped me have more credibility in my work and in my role as a state advocate and reflective leader*. *Endorsement provides a way to express and share a level of expertise not always recognized in non‐clinical settings. It helps others to better understand my level of training and specialization*. *It also provides more credibility as I am co‐located in a medical setting*. *Provides credibility in court*.
	151 (31)	Improved confidence and personal satisfaction	*Having my endorsement has helped build my confidence and professional identity, which directly impacts the care I provide*. *My endorsement has changed how I see myself and others around me. It has given me the ability to reflect and remain in the moment when working with young children*. *It has made me feel more accomplished in my profession*. *Endorsement has influenced my work by building my confidence in my ability to provide reflective supervision appropriately*. *Endorsement gave me the courage to reach outside my state and seek advanced opportunities*.
	114 (23)	No help/ created burdens	*Has not influenced or impacted. Would be doing the same job with or without endorsement* *It is also difficult and expensive to attain access to trainings to meet the endorsement requirements*. *It has required a lot more time while also trying to balance my actual work responsibilities* *It also is confusing how it has evolved over time and unclear why/when those that were originally endorsed at level 2 in (state) became something else*. *I had initially hoped for clinical endorsement but found the exam an unnecessary obstacle…It seems that exam was geared towards students that had completed coursework in Infant Mental Health, not for senior clinicians*. *Colleagues I know and respect well having even mentored me at times in the past have struggled to pass the exam which has increased my own anxiety about preparedness*.
What would you tell others about why they should earn Endorsement?	428 (48)		
	154 (36)	Support families and be a better provider	*It is the path to greater professionalism and provides more opportunities to experience collegiality. The knowledge leads to greater effectiveness and personal reward*. *It may surprise you with the ways it enhances your thinking, possibilities unfold*. *Endorsement provides clarity and a framework to support your work*. *It will enhance the work you're doing, help you in building a community of other endorsed providers and supplement your already strong credentials*. *I tell people it will guide you to being the best provider you can be*. *Earning endorsement will allow for professional growth that will impact your clients and your career*. *Endorsement is a way to demonstrate that you have competencies as a professional working on behalf of young children and their families*. *We acquire new skills and knowledge to work with our families and young children. We learn about child development, the importance of Safe—Positive—Nurturing Early Relationships in the development of healthy brains. We also learn about ourselves and how to better interact with children and families. We learn to respect differences and embrace diversity*. *Endorsed professionals are better prepared to support the early foundational development of babies and young children in the context of relationships*.
	124 (29)	Credibility	*Endorsement builds your professional identity and brand*. *Getting credit for the experience and training that you've already done*. *Endorsement is a process that acknowledges your training and hard work to date. It offers you, and others in your field, an opportunity to be recognized for the level of professionalism you bring to your work*. *Just as having a college degree validates one's expertise, I feel the Endorsement validates my knowledge and experience in the field of early childhood and specifically in infant mental health. The process helped me see myself as very competent in the field*. *Work on behalf of infants and young children is specialized work requiring providers to be highly skilled and trained. Endorsement lets us demonstrate that we've earned that*.
	114 (27)	Professional development and increase IECMH knowledge (RSC)	*You can gain more specialized knowledge of your work with young children and their families and if possible, RSC can give you new perspective on your work*. *The endorsement process and endorsement itself helped my self‐confidence in IECMH. Endorsement opens up further opportunities to support others through RSC and trainings that are crucial in supporting other IECMH clinicians*. *Endorsement helps you organize all of your disparate training, education, & working experiences into a coherent whole that matches your professional values, identity, & goals. It's a process, not an outcome; a journey, not a destination*. *Provides a pathway to maintain knowledge, skills, and growth*. *Applying for IECMH‐Endorsement provides a professional development opportunity/exercise to (re)organize your career information from an IECMH perspective. …Learning about possible IECMH Gaps can guide individual professionals toward strengthening knowledge and skills*. *Endorsement is a great opportunity to polish one's own knowledge and approach as an infant mental health practitioner*.
	102 (24)	Network and community	*Endorsement provides excellent opportunities to build capacities for ECMH and network with mindlike peers and leaders in this field* *Endorsement will set you up for multiple connections with like‐minded individuals. You'll have access to knowledge, resources, and opportunities that will propel your career with children because they actually care about the people*. *The trainings and the connections with others who hold similar interests are of value that cannot be easily categorized and labeled. I could not have predicted the directions my career path would take when I began to pursue this knowledge*. *It connected me to others in this field. I feel as though I am part of strengthening the field*. *It helps create a professional community and a shared language that allow us to be more cohesive and connected in this challenging, yet important work of early prevention and intervention*.
	93 (22)	Validates the profession and field	*The endorsement sets professional standards; the process of being endorsed provides a road map about skills, knowledge and experiences that are critical for the highly complex infant and early childhood field*. *The endorsement is a way to help regulate quality in the field*. *It is a way to professionalize the field and demonstrate knowledge and understanding of IMH*. *Earning Endorsement helps to further professionalize the family‐ and child‐serving workforce, increases reflective capacity, and can enhance feelings of self‐efficacy*. *I believe that early childhood mental health is a critically important field that often doesn't get the respect it deserves. This endorsement is a way to signal experience and expertise, both within the field and in surrounding areas of community mental health, education, advocacy and policy*. *Endorsement creates a shared collective of impeccably high standards and goals for our field (which is too often overlooked without the proper dignity and respect and admiration that it deserves)*.
	483 (52)	No response	“*N/A”*
What else would you like us to know?	340 (37)		
.	84 (25)	Endorsement needs more accessible support	*There seems to be a disparity between the inclusive intentions of endorsement outcomes, in terms of supporting infants in the context of diversity, equity and social justice, and the tight parameters and expectations towards those seeking endorsement, some of whom are very unlikely to meet compliance standards, through no fault of their own*. *I would advocate to review all categories and also work to have this credential recognized as an alternative license to be more valuable and credible. It would be more meaningful if the process wasn't tedious and didn't have barriers in place in order to have a more diverse group of endorsed professionals*. *The cost and time required to achieve endorsement need to be supported by the organizations of those applying*. *The language limits are important to note. Even though I was able to access, many of my colleagues could not as the requirements to apply are typically only available in English and Spanish and very little support for use of interpreters or translation*. *You need to be really determined to get the endorsement and a helping hand to complete the process. It was easy to give up if it wasn't for my RS‐C supervisor, that always encouraged me to keep working on it. Now I really want to complete the requirements for the level III*
	79 (23)	I'm grateful for Endorsement	*I like that the Endorsement process is becoming more transparent and more information is being shared to help applicants prepare for the application and the exam*. *I really appreciate the process as a self‐discovery journey–I realize how self awareness matters when working with others. I must understand the impact of relationships and recognize that everyone needs someone to hold a safe space for them*. *Endorsement as a journey has GREAT value. And I really appreciate the work that went into creating it & launching it & making it available outside of Michigan– AND I appreciate even more all the work that has recently gone into making it even better: more inclusive & accessible, less colonial/racist. There is always more work to do but great strides have been made in the past 4 years*.
	74 (22)	Wish Endorsement held greater value	*I basically applied for Endorsement as a personal/professional accomplishment and b/c it was paid for by my employer. I have received no compensation, promotion, etc. from my employer for being Endorsed. Besides for my co‐workers, only 1 other person has asked me about “the letters behind my name” and what that meant. It is really not acknowledged in our community*. *I was hoping the Endorsement would supplement my work with families, but the experience felt very separate instead of integrated into my practice as a marriage and family therapist. Because I navigated the process by myself, I might have misunderstood the intention/benefit of receiving Endorsement*. *I'm on the fence about renewing because there has been so much turmoil within our state AIMH. It is required for working in consultation here in (state) but if I leave consultation I'm not sure if I would continue to renew. I'm not sure I see the benefit other than employers seeing it as an area of expertise*.
	63 (19)	Limitations of RSC	*I wish there was financial support from the Infant Mental Health associations to pay for reflective supervision to support people towards endorsement so that the entire burden is not on the clinician themselves*. *I wish there was a better check and balance system for the providers of RSC. I have found through my own experiences that there are some very bad providers out there. RSC providers should have some sort of interview and vetting process aside from the exam*. *The requirement for RS starting next year will likely prohibit me from renewing. I would love to work towards providing RS to but I do not “qualify”*. *Reflective Supervision by someone really qualified is hard to get and expensive*. *There are many experienced and high quality practitioners that are amazing Reflective Consultants but because they are not vetted through the (association) or don't meet the education requirement it limits the accessibility to RSC. There are supervisors that have been in the field for years and are unable to support their supervisees with RSC because of the education requirement even though they have the training, practice, and experience more so than those that carry a masters*.
	59 (17)	Endorsement is valuable	*Endorsement is an excellent pathway in ensuring that young children birth to five are being served by competent professionals who has the training, skills and reflective capacity to hold all the complexities that they and their caregivers bring to relationship. We just cannot use a one size fits all with our young babies*. *I valued how detailed and comprehensive the process is for us*. *Now I know that if I meet someone who is endorsed at the mentor level (which is the one I know best) it means that person is well prepared in the topic*. *The care and thought that is taken to ensure the quality and fidelity to this process is one of the most unique and important aspects of ensuring the level of professionalization of our field*.
	58 (17)	Cost is a barrier	*The fee is really high and though I have the privilege of being able to pay for it, it was hard*. *We need to consider that some of the early childhood programs are nonprofits and may not have the funds to support endorsement. Also independent practitioners may also not have the extra financial resources to go through endorsement*. *It is a costly credential with no recognition of reimbursement or acknowledgement by insurance providers*. *Our setting, although they encourage endorsement, does not give financial assistance to achieve it. 3 classroom teachers were able to join a cohort of early childhood professionals funded by our state to earn endorsement. The teachers would not have attempted the endorsement without the state's funding*. *I wonder how making endorsement and renewal so specialized, costly, and cumbersome may make it inaccessible to many who work in low‐pay and busy childcare facilities*.
	571 (63)	No response	“*N/A”*

Percentages for endorsed themes were calculated based on the number of respondents who responded to the question, not including respondents who wrote “N/A.”.

Percentages for “No response” or “N/A” were calculated based on the entire sample of 911 participants.

#### 2.1.1 Question 1: How has Endorsement influenced your work?

This question was answered by 54 % (*n* = 494) of survey participants. A total of 20 themes were initially identified. Via the review process described above, the final number of major themes was reduced to four with 600 thematic responses. Three of the four final themes described a positive effect of earning Endorsement. The remaining theme was negative in valence, with responders reporting that Endorsement did not help at all or that it was a burden (23%). Exemplar responses for the most reported themes are included in Table 2.

##### 2.1.1.1 Theme 1a: Learning, growth, and improved knowledge

The most reported impact of Endorsement was increased knowledge, learning, and professional growth related to IECMH (36%; *n* = 178). Participants indicated experiencing a deeper understanding of IECMH concepts or a change in how they think about their work. Some participants also discussed realizing an increase in skills related to their ability to support the workforce.

##### 2.1.1.2 Theme 1b: Increased credibility and recognition as an expert

Thirty‐two percent of the respondents (*n* = 157) reported that earning Endorsement influenced their work by improving their credibility, including being seen as an expert or leader. Receiving this recognition was cited as important for workers in direct service, leadership, and advocacy roles. Some respondents reported that the Endorsement helps others recognize their level of training and specialization when they do not hold better‐known licenses or titles. Finally, participants indicated that Endorsement helps with their credibility in medical and legal settings.

##### 2.1.1.3 Theme 1c: Improved confidence and personal satisfaction

Thirty‐one percent of respondents (*n* = 151) noted that earning Endorsement increased their confidence in their knowledge, skills, or capacity to support others by providing RSC. Participants also cited feeling more confident in speaking about the work to others informally or formally through presentations and other professional opportunities. Some responders specifically discussed achieving personal satisfaction by being endorsed compared to tangible benefits such as higher pay.

##### 2.1.1.4 Theme 1d: No influence, did not help, was a burden

Twenty‐three percent of respondents (*n* = 114) reported that earning Endorsement had no positive influence on their work, citing that it did not result in benefits such as a better job or pay. Others indicated that because they were already engaged in IECMH practices, becoming endorsed did not change their daily work practices. Some reported that becoming endorsed was a burden, described as time‐consuming, with too many requirements, and not enough resources available to meet them, such as training. Participants also cited concerns related to the examination component of Endorsement. For example, participants cited concerns that the exam was geared toward clinicians rather than those from other sectors of the workforce.

#### 2.1.2 Question 2: What would you tell others about why they should earn Endorsement?

Four hundred and twenty‐eight participants (48% of the sample) responded to the second question. A total of 26 themes were initially identified. Via the review process described above, the major themes were reduced to five with 587 thematic responses. The percentage of responses assigned to themes ranged from 36% to 22%. Fifty two percent of responders (*n* = 483) left the question blank or wrote “n/a”. These responses were not included in the final calculations of themes. Exemplar responses for the most frequently reported themes are included in Table 2.

##### 2.1.2.1 Theme 2a: Better provider of services for babies, young children, and families

Thirty‐six percent of responders (*n* = 154) indicated that they would share that earning Endorsement led to being a better service provider for babies, young children, and families. Responses in this theme related to increased awareness, skills, or ways of thinking. Some participants mentioned an increase in self‐knowledge and a better ability to help families by promoting working with other professionals.

##### 2.1.2.2 Theme 2b: Credibility

One hundred twenty‐four responders (29%) indicated that they would tell others that the Endorsement credential provided them with personal credibility, understood as being seen as trustworthy and expert (Constantino et al., 2019). Participants cited receiving credit or acknowledgement for their training and experience, comparing it to having a college degree.

##### 2.1.2.3 Theme 2c: Increased IECMH knowledge

Multiple responders (27%; *n* = 114) reported that they would encourage earning Endorsement as it has increased their knowledge base of infants, young children, and families within the field of IECMH. Participants pointed to the process of preparing an application as a beneficial exercise in and of itself for organizing the knowledge that one has gained over time, as well as a support to identify gaps in one's experience. The requirement to participate in continuing education was also seen as valuable in maintaining growth, knowledge, and skills.

##### 2.1.2.4 Theme 2d: Networking and community

Another 24% of responders (*n* = 102) indicated that they find value in earning Endorsement because it exposed them to an IECMH community and allowed for networking and connecting with like‐minded professionals. These connections provided career opportunities for some participants.

##### 2.1.2.5 Theme 2e: Validates profession and field

Twenty‐two percent of respondents (*N* = 93) reported that Endorsement validates the profession and the field. Respondents indicated that earning an Endorsement was a way to set professional standards and increase respect for the field.

#### 2.1.3 Question 3: What else would you like us to know?

The third question received 417 responses by 340 participants (37% of the sample). Over 50% of respondents (*n* = 571) wrote “not applicable” or no answer was provided. These responses were not included in the major identified themes. A total of 22 themes were initially identified. Via the review process described above, the number of major themes was reduced to six. Two themes described a positive experience related to Endorsement. The remaining four themes offered constructive criticism of aspects of the Endorsement process/credential, suggesting a need for a change. Exemplar responses for the most reported themes from those who provided a response are in Table 2.

##### 2.1.3.1 Theme 3a: Endorsement needs more accessible support

One of the most salient themes from this open‐ended question was specific to the initial Endorsement process. Twenty five percent of respondents (*n* = 84) expressed confusion with the process and/or a need for more support throughout the process. These responses also included a need for more inclusion, diversity, equity, and anti‐racism throughout the Endorsement process. *Digging Deeper: De‐Colonizing our Understanding and Practice of Reflective Supervision Through a Racial Equity Lens* helps us define what these terms mean. Diversity‐informed practice is a system of beliefs and values guiding interactions within individuals, organizations, and systems of care. It acknowledges the intersectionality of oppression (e.g., racism, classism, sexism, ableism) and strives for inclusivity across all areas of practice, including teaching, research, policy, advocacy, and direct service (Thomas, Noroña & St. John, 2019). Anti‐racism is the active effort to oppose racism by advocating for and implementing changes in political, economic, and social systems. It involves recognizing and addressing the structural and systemic nature of racism, as well as challenging individual racist behaviors and their impacts (Race Forward, 2015).

##### 2.1.3.2 Theme 3b: Grateful for Endorsement

Twenty‐three percent of respondents (*n* = 79) indicated they were satisfied with the Endorsement process and the positive impact it had on their careers. Many of these respondents expressed a sense of pride in receiving their Endorsements.

##### 2.1.3.3 Theme 3c: I wish Endorsement held greater value

About 22% of respondents (*n* = 74) wanted more recognition in general for being endorsed. They wanted more clarification on why and how the Endorsement is valuable. Many of the respondents wished that the systems/organizations they were involved with understood the value of Endorsement.

##### 2.1.3.4 Theme 3d: Limitations of Reflective Supervision and Consultation (RSC)

Nineteen percent of responders (*N* = 63) reported concerns with limited access to reflective supervision and consultation required to attain some Endorsement types. Access problems included a lack of available supervisors/consultants and costs. In addition, requirements that the supervisor/consultant also hold an Endorsement were seen as a barrier to access.

##### 2.1.3.5 Theme 3e: Endorsement is valuable

Seventeen percent (*N* = 59) of responders described the value of attaining Endorsement. Comments included recommending earning Endorsement to others and stating that they would continue to renew their Endorsement.

##### 2.1.3.6 Theme 3f: Cost is a barrier

Seventeen percent of participants (*N* = 58) noted that costs to obtain and maintain the Endorsement, including dues for Association membership, exam fees, and payments for RSC, were prohibitive.

### 2.2 Quantitative results

#### 2.2.1 Demographic associations with qualitative themes

In our analyses of qualitative themes and race and ethnicity, significant associations were found in the *increased credibility and recognition as an expert* theme from the question, “What would you tell others about why they should earn Endorsement” (*X* ^2 ^= 14.69; *p* = 0.01; *V* = 0.13). Post hoc analyses revealed respondents who identified as Asian (*n* = 22, 2% of sample) were significantly more likely to cite increased credibility and recognition, as compared to all other races and ethnicities (all *ps *< 0.05). Significant associations were also found in the *validation of the profession/field* theme (*X* ^2^ = 15.58*; p* = 0.008; *V* = 0.14). Post hoc analyses revealed respondents who identified as Native American and American Indian (*n* = 8, 1% of sample) were significantly more likely to claim that Endorsement validates the professional field, as compared to all other races and ethnicities (all *ps *< 0.004). The theme that “*Endorsement was no influence, did not help, or was a burden”* was trending toward significance (*X^2^* = 9.74; *p *= 0.08; *V* = 0.11). Post hoc analyses revealed that respondents who identified as Native American and American Indian (*n* = 8, 1% of sample) were marginally more likely to claim that the Endorsement did not help them, as compared to other races and ethnicities (all *ps *< 0.06). No other significant associations were found between race and ethnicity and any other theme.

In our examination of the length of association establishment by themes, there were significant differences in Endorsement of *cost being a barrier* (*X* ^2 ^= 5.42; *p *= 0.02; Φ = 0.08). Post hoc analyses revealed that respondents who were affiliated with an association that was established over 10 years ago (*n* = 401, 47% of sample) were significantly more likely to identify cost as a barrier, as compared to respondents affiliated with a newer association (i.e., established less than 10 years ago; *n* = 446, 53% of sample). No other significant associations were found (all *ps *> 0.11).

When examining respondents’ years of experience in the workforce by theme, significant differences were found in the theme of “*validates the profession or field”* (*X* ^2 ^= 8.66; *p* = 0.03; Φ = 0.10). Post hoc analyses revealed that respondents with over 10 years of experience (*n* = 304, 36% of sample) were significantly more likely to cite validation of the field as compared to respondents with less than 10 years of experience (*p *= 0.004). Additionally, significant differences were found in the theme “*Endorsement needs more accessible support” (*e.g., to access reflective supervision; *X* ^2 ^= 11.03; *p* = 0.01; Φ = 0.11). Post hoc analyses revealed that respondents with over 10 years of experience were significantly more likely to report that Endorsement needs more accessible support as compared to those with less than 10 years of experience (*p* = 0.006). No other significant associations were found between years of experience and any other endorsed theme (all *ps* > 0.07).

In our examination of respondents' educational level by theme, significant differences were found in the theme of “*networking and community*” (*X* ^2 ^= 11.92; *p* = 0.02; Φ = 0.12). Post hoc analyses revealed that respondents with an advanced degree (e.g., Master's or Doctoral degree; *n* = 613, 72% of sample) were significantly more likely to report Endorsement's value of networking and community as compared to individuals with a Bachelor's degree or less (all *ps <* 0.06). Additionally, significant differences were found in the theme “*validates the profession or field”* (*X^2^* = 13.46; *p* = 0.009; Φ = 0.13). Post hoc analyses revealed that individuals with an advanced degree were significantly more likely to report that Endorsement validates the field as compared to individuals with a Bachelor's degree or less (all *ps *< 07). Finally, the theme, “*I'm grateful for Endorsement”* was trending towards significance (*X* ^2 ^= 8.58; *p* = 0.07; Φ = 0.10). Post hoc analyses revealed that individuals with an advanced degree were significantly more likely to report gratitude at obtaining Endorsement as compared to individuals with some college experience but no formal degree (all *ps *< 0.02). No other significant associations were found between educational attainment and any other endorsed theme (all *ps* > 0.16).

Finally, regarding examinations of the workforce sector and the Endorsement category by theme, there were no significant differences (all *ps *> .10).

## 3 DISCUSSION

Like earlier studies, the results of the survey found both benefits and barriers/challenges to earning Endorsement, confirming these findings in a broader sample. Findings of this survey are consistent with previous work that participating in Endorsement led to increased knowledge and skills (Krysik et al., 2019; McCormick & Eidson, 2020) and the opportunity to connect with others in the field (Dye et al., 2022). Barriers and challenges reported in the present survey were also similar to those reported by others. For example, the current results noted that financial costs are a burden, navigating the online application system (EASy) can be challenging, and it can be difficult to find a reflective supervisor, similar to results found by Krysik et al. (2019), who noted that it requires a strong commitment to see it through. Of note, identified themes had some similarities across the questions, suggesting consistency in participant viewpoints. Participants reported that Endorsement improved their knowledge and skills and made them better at their work. Unique to this study compared to previous work, participants reported feelings of gratitude for the Endorsement, acknowledging the benefit of the self‐discovery related to going through the process or that completing it resulted in personal satisfaction. This result, identifying an internal benefit, may relate to the emphasis on reflection and reflective practice that is evident in both the Endorsement process and infant and early childhood mental health practice broadly. It would be interesting to explore if participants also reported gratitude for more concrete benefits, such as feeling more competent or increased confidence, resulting in seeking additional training or certifications. Being recognized as more credible by others as an expert in the field was also noted across questions.

Although participants identified positives to attaining Endorsement, this is balanced with some concerns. Participants reported that Endorsement, while useful in some ways, did not typically yield tangible benefits or significantly influence their daily work or career trajectory. Many expressed concerns about the complexity and cost of the process, including financial burdens related to training, supervision, and renewal fees. It is noted that some participants highlighted the relative burden for those in the lower‐earning sectors of the workforce. There was a recurring need for more support and resources throughout the process, particularly in addressing financial and logistical challenges. Additionally, respondents raised concerns about the value and recognition of the Endorsement credential within professional systems, and some reported difficulties with the online application system and the transparency of the exam process.

Few differences were found for participants’ demographic characteristics in this sample. Those who identified as Native American/American Indian reported that Endorsement increased their credibility and elevated the credibility of the field. This group also reported that Endorsement did not help them personally, perhaps reflecting the differences between personal benefits like increased pay and less tangible benefits such as being viewed as more expert. Participants who had 10 years or more of experience were also more likely than those who had less than 10 years of experience to cite the value to the field while also expressing concern that more accessible supports (e.g., easier access to RSC) were needed.

We compared responses from participants who were members of associations established over and under 10 years. Members of the associations with longer longevity had more concerns about the financial burden. This may be a result of early funding of Endorsement through grants or state subsidization that was time‐limited, meaning that participants may have accessed support at first that was not available when time to renew participation.

Finally, we compared responses from participants by their highest educational attainment. It was revealed that individuals with an advanced graduate degree (i.e., Master's or Doctorate) were more likely to highlight Endorsement's value of networking, community, validation of the field, and report overall gratitude for the experience. This may be a result of increased attendance at conferences and other professional networking events that accompany graduate school experiences.

### 3.1 Strengths and limitations

The study demonstrates several strengths, including a large sample size with some diversity across participant characteristics (e.g., years of work experience, race and ethnicity, and workforce sector) as well as diversity in the associations represented (geographic location, years established). The research team members also have similar diversity; in addition, the research team includes both researchers and community members who are not researchers.

This study also has a few limitations to note that may have impacted our findings. This study focused on a sub‐sample that completed the open‐ended questions on the Endorsement survey. The current sample represented about 50% of the original sample participants, and we are unable to report if there were any demographic differences between open‐ended question responders and non‐responders. The entire survey was lengthy, and there was some overlap among quantitative and qualitative question content. It is possible that the length and repetition led some participants to discontinue the survey before completing all of the items, resulting in the lower response rate for the qualitative questions at the end of the survey.

We found no differences in participant workforce sectors. Respondents were allowed to select all applicable sectors. This resulted in many respondents who selected multiple sectors, which may have led to non‐significant findings in our association analyses (i.e., due to participants being counted in multiple groups). Thus, future studies may wish to alter survey data collection procedures to either have respondents select only one sector or select all that apply, but then identify their primary or main sector. This will allow for more direct comparisons across groups. We also found very few differences related to participant demographic characteristics. This may be due to the relatively limited diversity of the sample, particularly in race and educational attainment. As the workforce continues to diversify, it will be important to continue to assess for and respond to differences in preferences and needs related to training, RSC, and Endorsement. Additionally, while a large number of associations (*N* = 28), including international (e.g., Western Australia), were represented in the sample, many associations only had a limited number of participants respond, and thus we were unable to conduct cross‐site (i.e., state by state) and cross‐national (i.e., United States by Western Australia) analyses due to limited power. Future studies may wish to sample a larger number of international participants to determine if national differences exist.

Only individuals who completed the Endorsement process took part in this survey. Anecdotally, we know that some people begin the process and discontinue it. It is possible that people who do not complete the Endorsement would describe the process differently from those who have attained an Endorsement. In the future, it might be helpful to compare the experiences of those who had a hard time with the process, such as those who did not complete the Endorsement process, did not qualify for the preferred Endorsement type, or who earned Endorsement and did not maintain it, with those who had a smoother path.

## 4 IMPLICATIONS FOR NEXT STEPS

The findings from this survey highlight both the significant benefits and the challenges associated with earning Endorsement. While many participants reported improvements in their professional practice, credibility, and personal satisfaction, at the same time, they also hold clear concerns regarding the accessibility and recognition of the credential. Notably, issues related to financial and logistical barriers, the complexity of the process, and the perception that Endorsement does not always translate to tangible career benefits suggest areas for improvement.

Interestingly, participants from the longer‐established associations indicated more concern for financial barriers than newer associations. This finding may be explained by initial grants, state subsidization, and other support often being provided when an association is being established, but not sustained over time. Additionally, the findings could be driven by the experience of participants who are part of the early care and education workforce, which has historically been paid lower than other sectors (Lloyd et al., 2021).

The recurring need for more accessible support, particularly around reflective supervision and financial assistance, indicates that efforts should continue to make the Endorsement process more equitable and inclusive. Some changes have already been implemented in the Endorsement process that may address identified concerns. For example, since this survey was conducted, the Endorsed Reflective Supervisor designation was developed and launched in January 2024. This add‐on credential allows current endorsees to be recognized as eligible providers of qualifying hours of RSC. This new Endorsement eliminates the need for an examination for this group, as it was determined that the exam did not effectively assess competence in RSC. The creation of this designation has significantly expanded the pool of professionals able to offer RSC, particularly within the prevention and early intervention scope of practice. By increasing the types of providers eligible to become supervisors, increased access to RSC is created. Moving forward, it will be important to evaluate whether this designation successfully increases access to reflective supervision and if quality is maintained in the supervision experience.

Future research could also explore the experiences of those who begin but do not complete the Endorsement process to identify additional barriers and inform targeted solutions. Additionally, investigating the long‐term career impacts of holding the Endorsement, particularly in diverse professional settings, would provide valuable insights into the credential's true value in different contexts.

By addressing identified gaps and continuing to refine the Endorsement process, future studies could play a critical role in enhancing the accessibility and perceived value of this important credential, ultimately contributing to a more competent and supported infant and early childhood mental health workforce.

## CONFLICT OF INTEREST STATEMENT

The following authors are endorsed and do paid work that requires maintaining Endorsement: Tomlin, McCormick, Battles, Mober, Gutierrez‐Albrecht, Pariborzi, and Morelen.

## Data Availability

The data that support the findings of this study are available on request from the corresponding author. The data are not publicly available due to privacy or ethical restrictions.
